# Surface roughness effect on fatigue strength of aluminum alloy using revised stress field intensity approach

**DOI:** 10.1038/s41598-021-98858-0

**Published:** 2021-09-29

**Authors:** Bingfeng Zhao, Jiaxin Song, Liyang Xie, Zhiyong Hu, Jianpeng Chen

**Affiliations:** 1 Northeastern University, Shenyang, 110819 People’s Republic of China; 2 Key Laboratory of Vibration and Control of Aero-Propulsion System Ministry of Education, Shenyang, 110819 People’s Republic of China

**Keywords:** Metals and alloys, Mechanical properties

## Abstract

The fatigue strength of a component is known to highly depend on its surface quality, and it is thus necessary to develop a reliable and appropriate mathematical model for fatigue strength assessment that consider the effect of surface roughness. In this paper, different underlying physical mechanisms of the roughness effect at different regions of specimens were studied by fatigue testing of 7N01 aluminum alloy. For a quantitative analysis of the surface roughness effect, a revised stress field intensity approach for a fatigue strength assessment of microsized notches was proposed as a theoretical support. In the new model, a new form of weight function was built to adapt the characteristics of microsized notches. In addition, the effect of the field radius was fundamentally weakened on solution of the stress field intensity and the difficulty of fatigue failure region definition in the traditional method was overcome correspondingly in the proposed model, which made the calculated field strength accurate and objective. Finally, to demonstrate the validity of the revised approach quantitatively, specimens with conventionally sized notches were subjected to stress field intensity calculations. The results showed that the revised approach has satisfactory accuracy compared with the other two traditional approaches from the perspective of quantitative analysis.

## Introduction

It is known that the fatigue strength of component depends largely on its surface quality, which is the key content and core technology to improving the surface quality of the component during the design process^1^. In engineering applications, the parameter of surface roughness is employed to describe the surface quality of the component. It is an important index used to reflect the microgeometric morphology of the component surface, relating to the microgeometric characteristics of small spacing and peak-valleys on the material surface. The effect of surface roughness can reduce the fatigue strength of components and can even cause disastrous consequences when the applied load level is below the fatigue limit of the material. During the component production process, it is almost infeasible to avoid the effect of surface roughness, but it can be controlled during machining. As a consequence, for the purpose of improving the fatigue properties of the component as much as possible, accessing the fatigue strength of components with different surface roughnesses is a particularly important task. However, contradictions between the economic feasibility of the high cost of micro level analysis and high accuracy requirements of fatigue strength evaluation can lead to an unsatisfactory situation. Thus, appropriate mathematical models for fatigue strength assessment that consider the effect of surface roughness are required to clarify the contradictions^2^.

Considering that fatigue failure generally appears on the surface of a material, it is commonly accepted that the surface roughness has an enormous effect on the fatigue strength of the component. The surface roughness of the component can imply that the surfaces of the material are no longer perfectly flat, which will promote grain slip and crack initiation at the surface of the material. In other words, surface roughness has a significant impact on the underlying physical mechanisms of crack initiation for the components^3^. In conventional approaches, the effect of surface roughness is commonly accounted for by introducing an empirical reduction factor that can modify the fatigue strength limit of the material. Directed against AISI 4140 steel, Taylor^4^ compared the fatigue strengths of four kinds of components with different machining methods and concluded that the fatigue strength of the material decreased with increasing surface roughness. Similarly, Itoga^5^, Hatamleh^6^ and Bagehorn^7,8^ studied the different cracking initiation mechanisms and S–N curves of Ni–Cr-Mo steel, AA-7075-T651 alloy and Ti-6Al-4 V with different surface roughnesses. The results showed that surface roughness was a significant factor leading to a short life regime and that fatigue properties decreased when the surface roughness of the component increased, similar to the conclusions obtained by Taylor and Arola. A lot of experiments have been conducted by Silva^9^ and Wang^10^ in related fields, by which the variation regularity of fatigue properties with different surface roughnesses was studied, as well as the corresponding analytical methods.

According to the results of the research, current engineering guidelines, as well as software packages, are known to highly depend on the empirical reduction factor contrived by Lipson and Noll in the 1950s^11^. These parameters were classified on the basis of different machining procedures, which were related to the stress strength of the metallic material available at the time. In recent years, engineers have faced a broader range of metallic materials that can hardly categorized according to the stress strength. Furthermore, for quantitative analysis of the surface roughness effect, some appropriate mathematical calculation methods should be employed to overcome the aforementioned matters^12,13^. First, the effect of surface roughness should be represented starting from the analysis of the surface stress state caused by the surface asperities of the material, which can allow modern theories for fatigue crack initiation to be used, along with the basic fatigue parameters of the material^14,15^. At present, finite element analysis (FEA) is accepted as a mainstream approach to the near-surface stress state of components. During finite element analysis, treatment methods of the surface topography are key content and core technologies for analysis of the surface stress state. Aanrews^16^ and Misra^17^ proposed simplifying the surface micronotch topography to an approximate semielliptical notch, which is currently a commonly used simplification method. To evaluate the stress concentration state on the surface of high-strength steel, an ideal sinusoidal micronotch was used by Arola^18^ and Embrechts^19^ to simulate the complex topography of the material surface. Based on basic assumptions, effect of surface quality on the fatigue of life AISI 4130 CR steel was assessed in terms of the stress concentration effect. The Arola-Ramulu model was proposed and the comparison result with the Neuber formula was given based on the test data of AISI 4130 CR steel. After the analysis result of the surface stress state is presented, a fatigue strength assessment model of the notch is needed to solve the following problem.

The fatigue strength assessment of notchs has attracted much attention from many global famous scholars and a lot of strength assessment models have been built in recent decades^20–24^. In fatigue analysis for a notched component, although the most common approaches used in engineering applications, such as the nominal stress criterion^25,26^ and local stress-strain criterion^27,28^, are straightforward and efficient, many studies shown that their reliability and accuracy of them are unstable under some conditions^29–31^. For this reason, many other methods have been proposed. Since Beltrami's first work, the concept of strain energy density has been widely used to evaluate the fatigue performance of smooth and notched structures^32,33^. In particular, energy based standards show superior ability in unifying micro and macro experimental evidence and establishing life prediction models^34^. In the field of material research, optical microscopy and scanning electron microscopy have been used as a technical means to reveal the damage evolution law of material microstructure under different loading conditions, to analyze the fatigue problem more mesoscopically, which has achieved good results in the application of some special alloys^35,36^. In recent years, with increasing attention given to the safe service performance of engineering components, stricter requirements have been put forward for the prediction accuracy of structural fatigue life and the safety margin of anti-fatigue design. Considering this situation, scholars in the industry have gradually expanded the research method of notch fatigue from deterministic analysis to probabilistic modeling, by which the probabilistic fatigue method has emerged to better meet the engineering needs of designers and users^37–39^. Although the above methods have been widely used, the analysis results are still unsatisfactory on many occasions. This is largely because fatigue failure for components with stress concentrations will not occur in a single peak point of stress, but in a certain high stress region with the effects of a stress/strain gradient^40^. From this point of view, the critical distance theory evaluates the fatigue behavior of materials by introducing the concept of effective stress in the stress concentration zone, which has been used to predict the fatigue life of notchs under different materials and loading conditions^41,42^. Furthermore, Yao^43^ proposed a new equivalent stress calculation criterion, named the stress field intensity approach which can consider the effects of the loading condition, notch shape and size on the fatigue characteristics, to calculate the fatigue damage parameter of the notch according to the fatigue failure mechanism of the material. In the current criterion, instead of the stress peak used in traditional approaches, the effective damage parameter was set to be the stress field intensity for the high stress region^44^. In recent years, corrections of the fatigue failure region and weight function for the new criterion have been made by Shang^45^ and Tang^46^ respectively, and its accuracy has also been verified in many ways. As the definition of the fatigue damage region is complicated for the calculation of the stress field intensity, this new approach is not commonly promoted in engineering applications.

During the analysis of the surface stress state caused by the surface asperities of the material, the global asperity of the specimen surface was referred to in the same position. From the test point of view, there is no objection to this process for a specimen with centrosymmetrical cross sections in the gauge position. For specimens with edges and corners, however, considering the fact that fatigue failure generally appears at its edge or corner, it is obvious that the surface roughness at the edge or corner has a greater influence on the fatigue strength of the specimen than that of the internal surface. In this case, it's necessary to distinguish the effects of surface roughness existing at the edge/corner and internal surface. The main aim of the study is to analyze the different underlying physical mechanisms of the roughness effect at different regions of specimens. In terms of experiments, aluminum alloy specimens, with different regional characteristics and surface qualities, are subjected to fatigue tests. In addition, a revised stress field intensity approach is proposed as the theoretical support for fatigue strength assessment of microsized notches, in which the definitions of the weight function and fatigue failure region are revised. The effect of the field radius is fundamentally weakened to solve the stress field intensity by the proposed model. The difficulty of fatigue failure region definition in the traditional method is overcome correspondingly, which makes the calculated field strength accurate and objective.

## Experimental program for specimens with different roughness

To study the difference in roughness effects at different regions of specimens, solid bar specimens and planar plate specimesn with different regional characteristics and surface qualities were designed and tested in fatigue experiments.

### Materials and specimens

The 7-series aluminum alloy is a commonly used aeronautical aluminum, which has high strength and fracture toughness, good hot working performance, excellent welding acceptance and robust corrosion resistance. The 7N01 aluminum alloy is particularly used in aerospace and rail transit industries. Thus, a 7N01 aluminum alloy is employed in our fatigue experiment for the fatigue failure simulation of components in major and complex mechanical equipment, such as aerospace and rail transit equipment. The chemical compositions of the 7N01 aluminum alloy, obtained from the material manufacturer are shown in Table 1.

**Table 1:** Chemical compositions of the tested material (wt.%).

Zn	Mg	Cu	Mn	Cr	Ti	Zr	Si	Fe	Al
4.80	1.80	0.15	0.55	0.24	0.17	0.22	0.23	0.35	Balance

**Figure 1:**
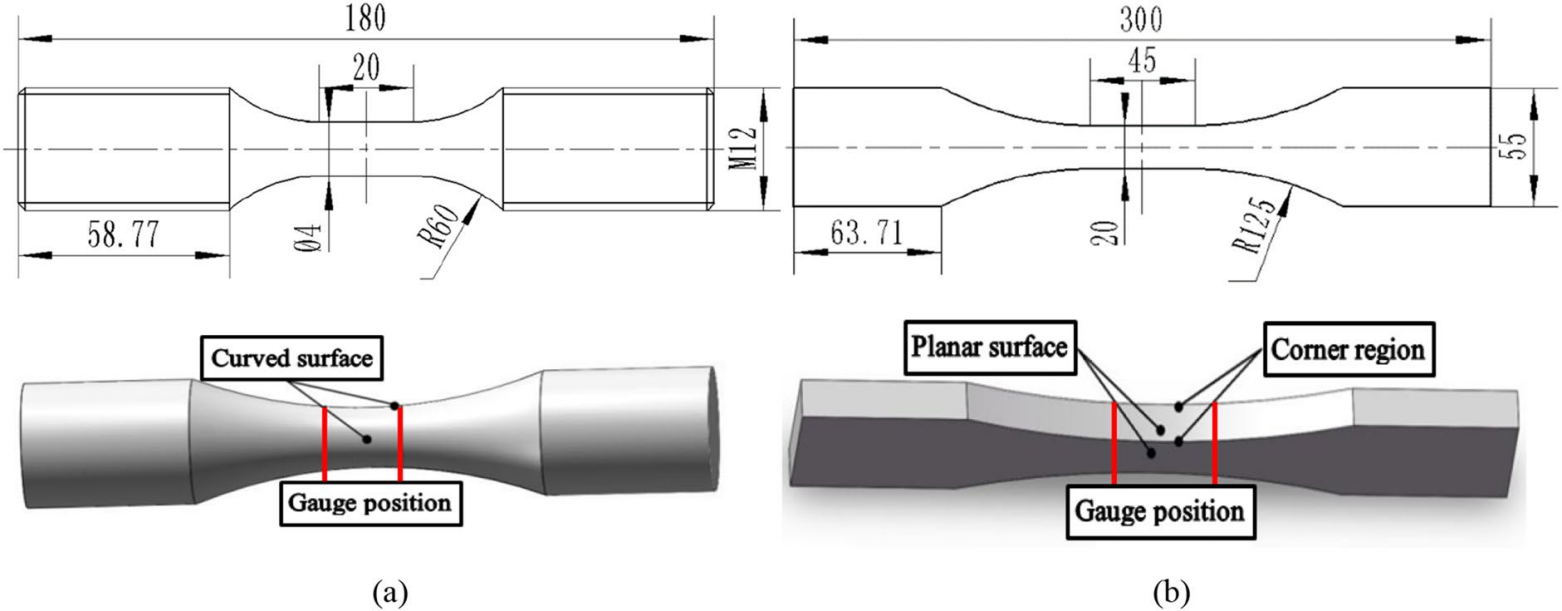
Geometry and dimension of the tested specimens: (**a**) solid bar specimen, (**b**) planar plate specimen. (Unit: mm).

Two types of specimens were tested in the fatigue experiment, including solid bar specimens and planar plate specimens, as shown in Fig. 1. The planar plate specimen had two types of specs with two different thicknesses, 2 mm and 10 mm. To reduce the dispersion in the experiment, specimens were all selected from the same batch and prepared along the rolling direction of aluminum sheets in accordance with the relevant standards.

Defined on the different regional characteristics of the specimen, the solid bar specimen contains a curved surface only in its gauge position and the planar plate specimen contains two kinds of regions in its gauge position including the planar surface and corner region, as shown in Fig. 1. To demonstrate roughness effects at different regions of the specimen, 8 groups of specimens with different roughness combinations were subjected to fatigue testing, as shown in Table 2. For ease of polishing the corner region, the outer corner of the planar plate specimen was ground to a chamfer with a size of 0.1 mm. In addition, it was unavoidable that a small part of the internal surface around the corner was categorized as the corner region during the grinding process of the planar plate specimen.

**Table 2:** 8 groups of specimens with different roughness combinations.

Specimen	Planar surface *Ra*/μm	Corner region *Ra*/μm	Curved surface *Ra*/μm	Specimen number
Planar plate specimen (2 mm)	1.6	1.6	–	A-1
	1.6	0.4	–	A-2
	0.4	0.4	–	A-3
Planar plate specimen (10 mm)	1.6	1.6	–	B-1
	1.6	0.4	–	B-2
	0.4	0.4	–	B-3
Solid bar specimen	–	–	1.6	C-1
	–	–	0.4	C-2

### Experimental procedure and results

**Figure 2:**
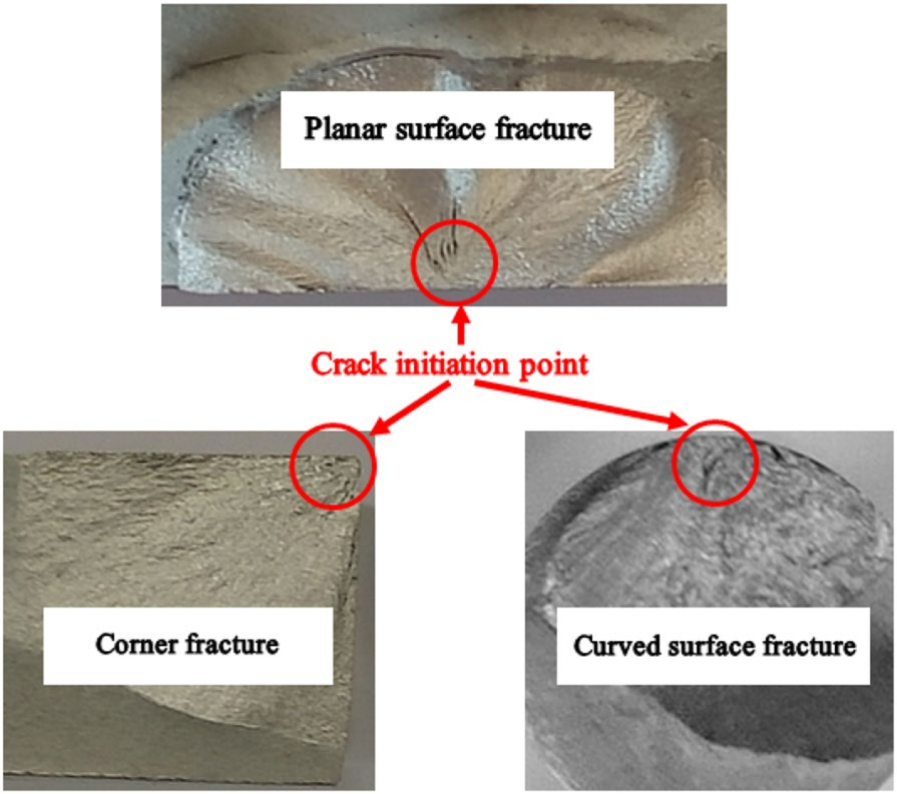
Fracture morphology comparison of different fracture modes.

Fatigue experiments for planar plate specimens and solid bar specimens were carried out on QBG200 and QBG100 fatigue testing machines (electromagnetic resonance high frequency test system). The fatigue loading was a sinusoidal wave with frequency and ratio of 150–180 Hz and -1, respectively. Other experimental details followed the guidance of ISO 12107: 2003 (eqv GB/T 24176-2009 in China). For the fatigue limit test of the specimens, an up-and-down test method was conducted in the experiment with an initial stress level of 190 MPa. The fatigue limit of the specimen was defined to be the stress level corresponding to the "infinite life" (10E7 cycles). The test continued until the specimen overflowed (the specimen maintained an intact shape after 10E7 loading cycles) or fractured. In addition, three kinds of fracture modes were defined, named corner fracture, planar surface fracture and curved surface fracture, according to the fatigue crack initiation location, as shown in Fig. 2. The stress levels and experiments for the 8 groups of specimens are listed in Table 3. In Table 3, the sign ‘-’ indicates that the specimen overflows after 10E7 loading cycles; the signs ‘+’, ‘×’ and ‘※’indicates that the fatigue crack initiates from the corner region (corner fracture), planar surface (planar surface fracture) and curved surface (curved surface fracture), respectively. In theory, it is obvious that the initial crack is more likely to initiate at the gauge position of the specimen. However, due to the existence of extra machining defects on the specimen surface, there were few initial cracks initiating from the transition arc or clamping position, which was regarded as fortuitous event in our study. During the compilation of experimental result statistics, only regular events with crack initiating from the gauge position of the specimen were taken as valid data, at 120 specimens in total, to maintain the accuracy of the results.

**Table 3:** Stress levels and experimental results for 8 groups of specimens (Unit: MPa).

No	A-1	A-2	A-3	B-1	B-2	B-3	C-1	C-2
1	190/+	190/−	190/−	190/×	190/−	190/−	190/−	190/−
2	182/−	198/−	198/−	182/+	198/+	198/−	198/※	198/−
3	190/+	206/×	206/−	174/+	190/−	206/−	190/※	206/−
4	182/+	198/−	214/+	166/−	198/×	214/−	182/−	214/−
5	174/+	206/−	206/−	174/−	190/−	222/−	190/−	222/※
6	166/−	214/−	214/−	182/+	198/−	230/−	198/−	214/−
7	174/+	222/×	222/+	174/+	206/×	238/×	206/※	222/−
8	166/−	214/+	214/−	166/−	198/−	230/+	198/※	230/−
9	174/+	206/−	222/+	174/+	206/+	222/×	190/※	238/※
10	166/−	214/−	214/+	166/−	198/−	214/+	182/※	230/※
11	174/−	222/+	206/−	174/−	206/−	206/−	174/※	222/※
12	182/−	214/−	214/−	182/×	214/−	214/+	166/−	214/※
13	190/−	222/×	222/−	174/+	222/−	206/−	174/−	206/−
14	198/+	214/+	230/−	166/+	230/−	214/+	182/−	214/−
15	190/+	206/+	238/×	158/−	238/×	206/+	190/−	222/−

**Figure 3:**
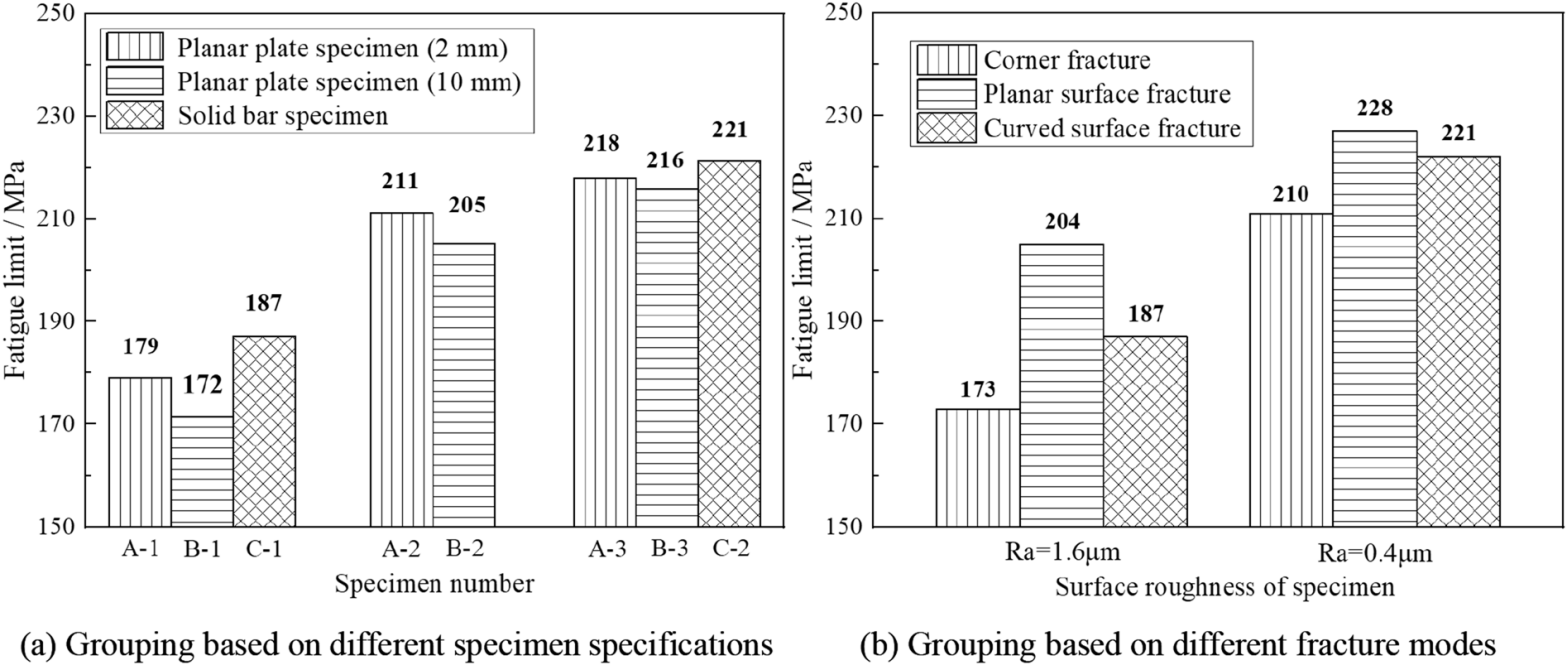
Fatigue limits of 8 groups specimens of based on different grouping methods.

**Figure 4:**
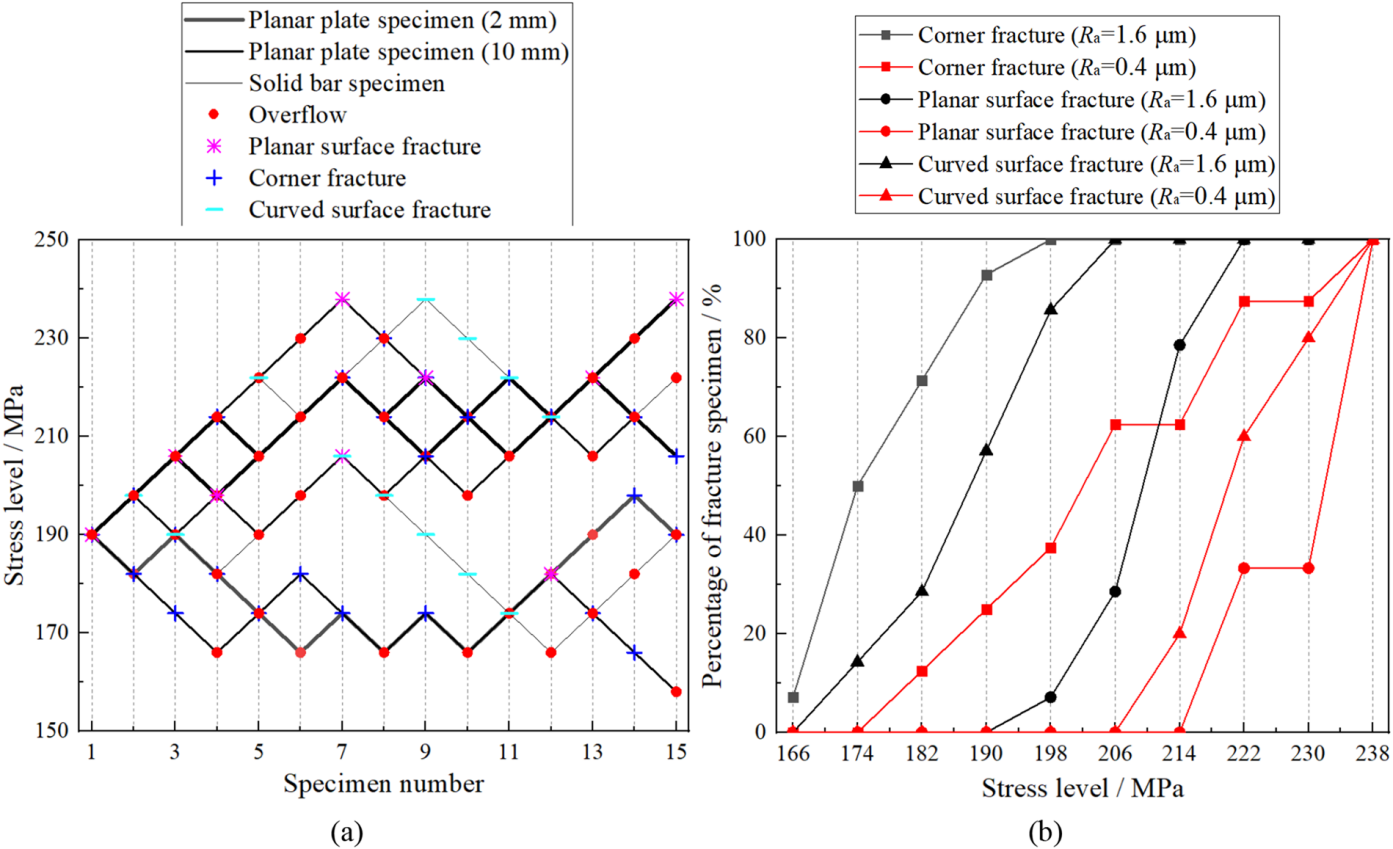
Up-and-down chart of the 8 groups of specimens (**a**) and distributions of the three kinds of fracture modes (**b**).

The matched pair technique can be employed to calculate the fatigue limit of the specimen, by which the mean values and standard deviations of the 8 groups of specimens are illustrated in Fig. 3a. In addition, to contrast the roughness effects at different regions of the specimen, the fatigue limit results calculated based on different regional characteristics and surface qualities are illustrated in Fig. 3b. According to Fig. 3, the following conclusions can be drawn:

1. By comparing the fatigue limit on various criteria in Fig. 3, it is obvious that surface roughness is a significant influencing factor for the fatigue strength of the specimen. When the surface roughness (*Ra*) decreases from 1.6to 0.4 μm, the fatigue limit of the specimen increases. This can be interpreted as the surface micronotch topography becoming smooth with decreasing surface roughness, which will promote grain slip and crack initiation at the surface of the specimen.
2. Based on the polished region, as calculated in Fig. 3a, the increased percentage of planar plate specimen fatigue limit by polishing the corner region and planar surface can be obtained at 18.5% and 4.3%. Considering that the fatigue limit of the planar plate specimen in Fig. 3a can be affected by different fracture modes, the increase percentage of the fatigue limit for a single fracture mode can be calculated in Fig. 3b to be 21.4% and 11.8% for corner fracture and planar surface fracture, respectively. From the calculation results, an intuitive conclusion can be obtained that there exists enormous variety among the roughness effects at different regions on planar plate specimen, especially for the effects on different fracture modes, which means the roughness effect at the corner region is much more prominent than that of the planar surface for the fatigue limit of planar plate specimen.
3. The fatigue limit of the specimen increases in turn corresponding to corner fracture, curved surface fracture and planar surface fracture. Differences among the three fracture modes will be obscure or unclear with decreasing surface roughness, as shown in Fig. 3b. To describe this conclusion in more detail, the experimental results for 8 groups of specimens are statistically analyzed with an up-and-down chart, as shown in Fig. 4a. Furthermore, the percentage of fracture specimens for the three kinds of fracture modes under different stress levels is illustrated in Fig. 4b. In Fig. 4, it is obvious that, in view of the overall situation, the stress levels of corner fracture, curved surface fracture and planar surface fracture increase in turn at the same percentage of fracture specimen. In other words, the possibilities of corner fracture, curved surface fracture and planar surface fracture decrease in turn under the same stress level, which means that the stress concentration factors of the same micronotch on the corner region, curved surface and planar surface decrease in turn under the same nominal stress.

## Evaluation of the fatigue damage parameter for the effects of roughness

The stress state of the fatigue specimen needs to be clarified for the calculation of the effective damage parameter. Some analytical and numerical approaches can be used to analyze the stress/strain state in simple cases. However, it is almost infeasible to launch the analysis by using these approaches in engineering practice because of complexity and poor adaptability conditions, where the FEM can be chosen as another available method for a stress state analysis of surface micronotches.

**Figure 5:**
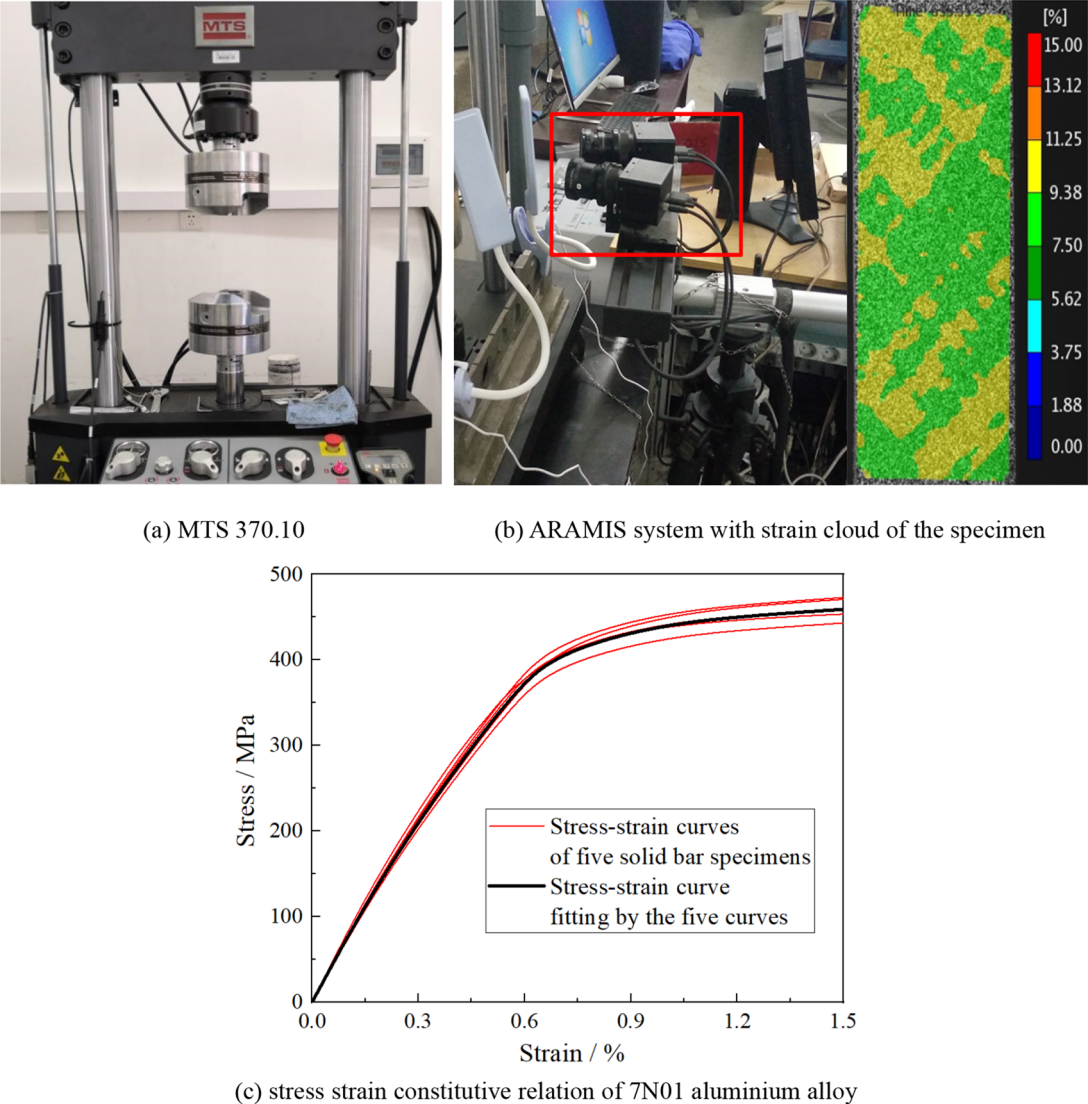
Experimental equipment (**a**, **b**) for uniaxial tensile experiment and the tested stress–strain curve of 7N01 aluminium alloy (**c**).

In our study, ABAQUS was chosen to calculate the stress distribution around the surface micronotch of the specimen, where the stress strain constitutive relation of 7N01 aluminum alloy is needed. In our study, solid bar specimen (Fig. 1a) was tested by a uniaxial tensile experiment at room temperature to obtain the stress strain constitutive relation of 7N01 aluminum alloy. An MTS fatigue testing machine (hydraulic servo test system MTS370.10) with the assistance of the ARAMIS system was used to perform the uniaxial tensile experiment, as shown in Fig. 5a,b. The simplified stress–strain curves of five specimens were obtained by GB/T 228-2002 (eqv ISO 6892: 1998) and the stress strain constitutive relation of the 7N01 aluminum alloy needed in the elastic-plasticity FEM analysis should be fitted by the five specimens, as shown in Fig. 5c.

**Figure 6:**
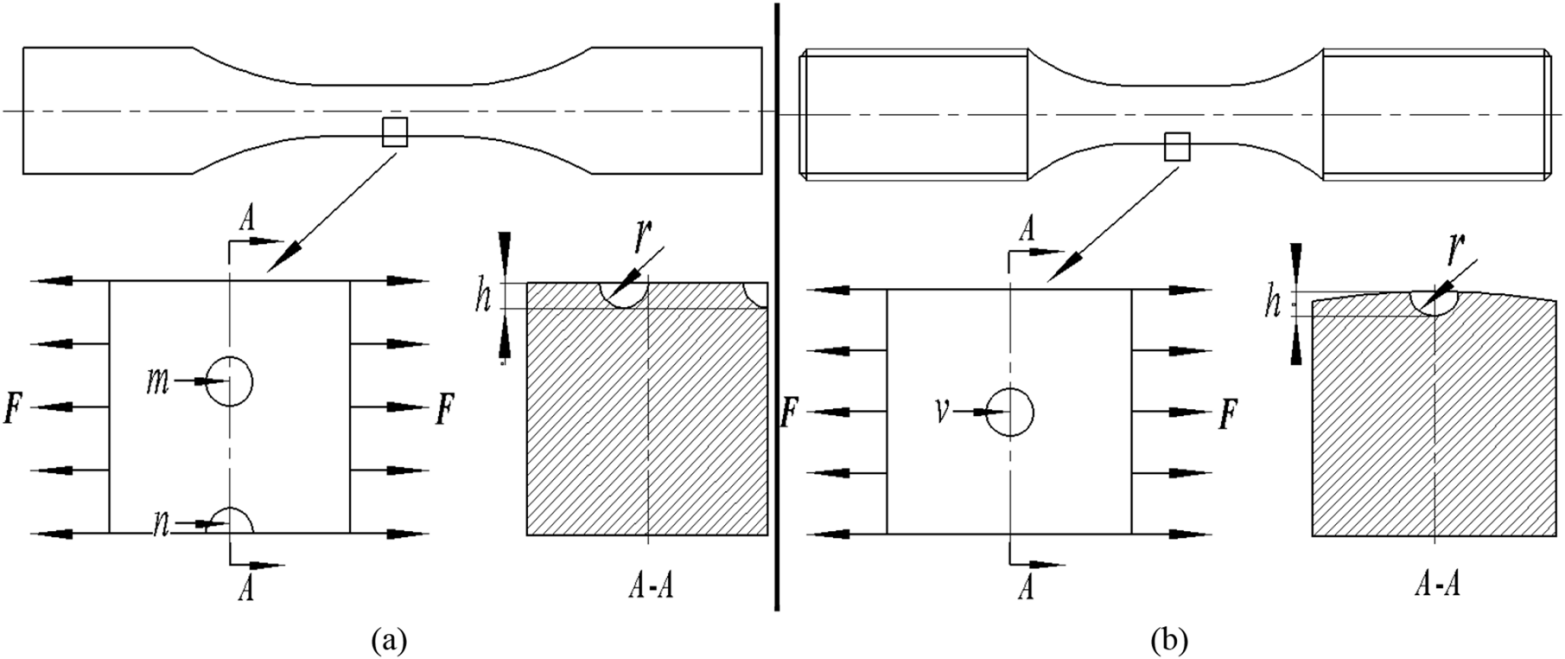
Sampling diagram of block element for FEM analysis: (**a**) Planar plate specimen; (**b**) Solid bar specimen.

Because there are orders of magnitude differences between the size of the surface roughness and that of the specimen involved in this test, it is almost infeasible to describe the surface micronotch topography using a full-size specimen. For this reason, a block element was selected from the gauge position of the specimen for FEM analysis and the block elements for the solid bar specimen and planar plate specimen are shown in Fig. 6. The size of the block element is 100*100*100 μm, by which the loading force can be calculated by

1 $$F = \frac{{\sigma_{{\text{s}}} }}{100}$$

where *F* is the loading force for the block element (Unit: N). *σ*_*s*_ is the nominal stress of the gauge position on the specimen (unit: MPa). The surface micronotches on the corner region, planar surface and curved surface are represented by *n*, *v* and *m*, respectively. In our study, the surface micronotch was simplified as a spherical notch. The effect of surface roughness on the fatigue strength of specimens is usually approached in terms of the stress concentration, which means that it is reasonable to describe the relationship between them in terms of the stress concentration factor *K*_*t*_. According to Reference^47^, the stress concentration factor for a single micronotch subjected to uniform tension can be calculated by the notch root radius (*r*_root_) and notch depth (*h*)

2 $$K_{t} = 1 + 2\sqrt {\frac{h}{{r_{{{\text{root}}}} }}}$$

**Figure 7:**
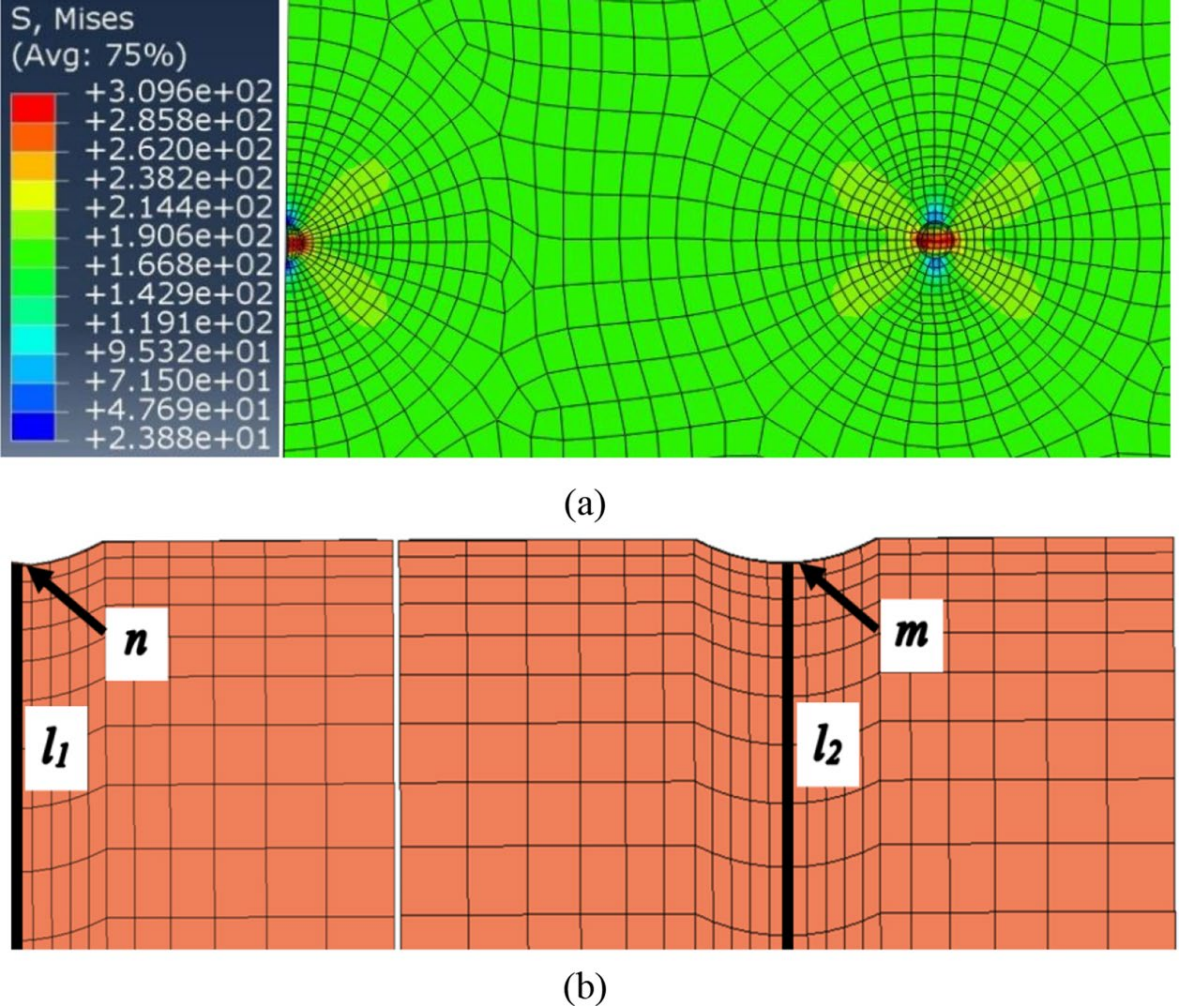
Computational block element stress nephogram: (**a**) Global stress nephogram; (**b**) Symmetrical line of micro-notches *m* and *n*.

From Eq. (2), it is obvious that the surface roughness effect is relevant to the notch root radius and notch depth. In most cases, there is a complex coupling relationship between the two parameters, and it is thus difficult to take both parameters into account in force analysis. Even now, it remains unclear exactly how the geometrical dimensions of the small notches introduced in the FE model can be determined, which means it is difficult to perform a quantitative analysis of micronotches through the FE method. Considering the purpose of qualitative analysis in this paper, some necessary simplification work should also be done before the finite element analysis. In this paper, the arithmetic mean deviation of contour *Ra* (instead of average width of contour cells *Rsm*) was selected as a parameter to describe surface roughness, in which the characterization of roughness by notch depth is more representative than notch radius. For this reason, a qualitative analysis of the variable notch depth, described by the average notch depth of the surface micronotch (*Ra*), was carried out only to determine the effect on the fatigue strength, while the value of the notch root radius was regarded as a constant value, and a value of 5 μm was determined by the statistical law of the surface micro profile. In this case, the results of FEM analysis cannot be considered completely accurate. However, as the change in notch root radius would cause an overall change in stress history, the tendency of qualitative analysis results based on variable notch depth would hardly change much even if notch root radius was taken as another value. In other words, the tendency of qualitative analysis results based on variable notch depth, which is required in subsequent analysis, can be obtained in the notch root radius assumption. Taking the planar plate specimen as an example, by using an 8-node isoperimetric element with 8 integration points, a total of 77,800 elements and 82,212 nodes were used for the block element of the specimen. The stress strain constitutive relation and constraint condition of the specimen are shown in Figs. 5 and 6, respectively. The finite element analysis result with a surface roughness *Ra* = 1.6 μm is illustrated in Fig. 7 when the stress level is 190 MPa.

### Two kinds of traditional methods

#### Maximum stress criterion

**Figure 8:**
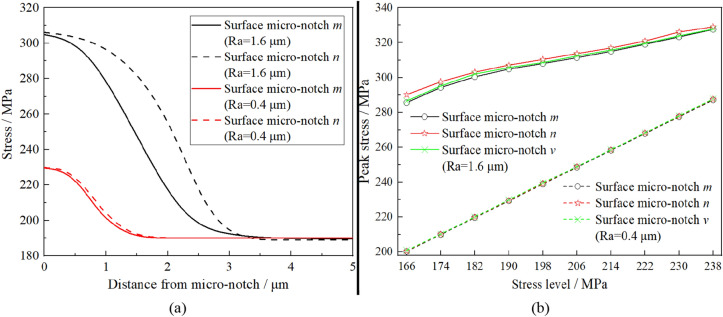
Stress states on the symmetrical line of different micro-notches (**a**) and peak stresses under the different stress leveles (**b**) with different surface roughness.

For maximum stress criterion, the peak stress at surface micro-notch is taken as fatigue damage parameter for fatigue performance assessment. Similarly, the finite element analysis result with the surface roughness *Ra* = 0.4 μm can be obtained when the stress level is 190 MPa. The stress states on the symmetrical line of micronotches *m* and *n* (*l*_*2*_ and *l*_*1*_ in Fig. 7b) with different surface roughnesses are illustrated in Fig. 8a. In addition, the peak stresses of different micronotches under the different stress levels are illustrated in Fig. 8b. In Fig. 8, it can be concluded that there is almost no difference among the peak stresses of micronotches *m*, *n* and *v* under the same stress level, which can not explain the phenomen in the fatigue test, i.e., the three conclusions in Section “Experimental procedure and results”. Therefore, a fatigue damage parameter calculation model is needed for further statistical analysis of the stress states around different surface micronotches.

#### Traditional stress field intensity approach

Considering that the fatigue damage always occurs in a certain region, instead of a single point with the peak stress, a new damage parameter, including material parameters, peak stress and stress gradient near the notch root, was proposed in the stress field intensity approach

3 $$\sigma_{FI} = \frac{1}{V}\int\limits_{\Omega } {\sigma_{r - \theta } \times } \varphi (r - \theta ){\text{d}}v$$

4 $$\varphi (r - \theta ) = 1 - \left| {\frac{1}{{\sigma_{\max } }} \times \frac{{{\text{d}}\sigma_{r - \theta } }}{{{\text{d}}r}}} \right|r(1 + \sin \theta )$$

where *σ*_*FI*_ is the stress field intensity. Ω is the fatigue failure region near the notch root. *V* is the volume or area of Ω. *θ* is the deviation angle from the reference point. *r* is the distance from the notch root. *φ*(*r*-*θ*) and *σ*_*r*-*θ*_ are the weight function and equivalent stress of the reference point with deviation angle *θ* and distance *r*. *σ*_max_ is the stress of the notch root (peak stress). To date, it has not been possible to quantitatively relate the fatigue failure region to the fatigue damage mechanism^43,44^.

**Figure 9:**
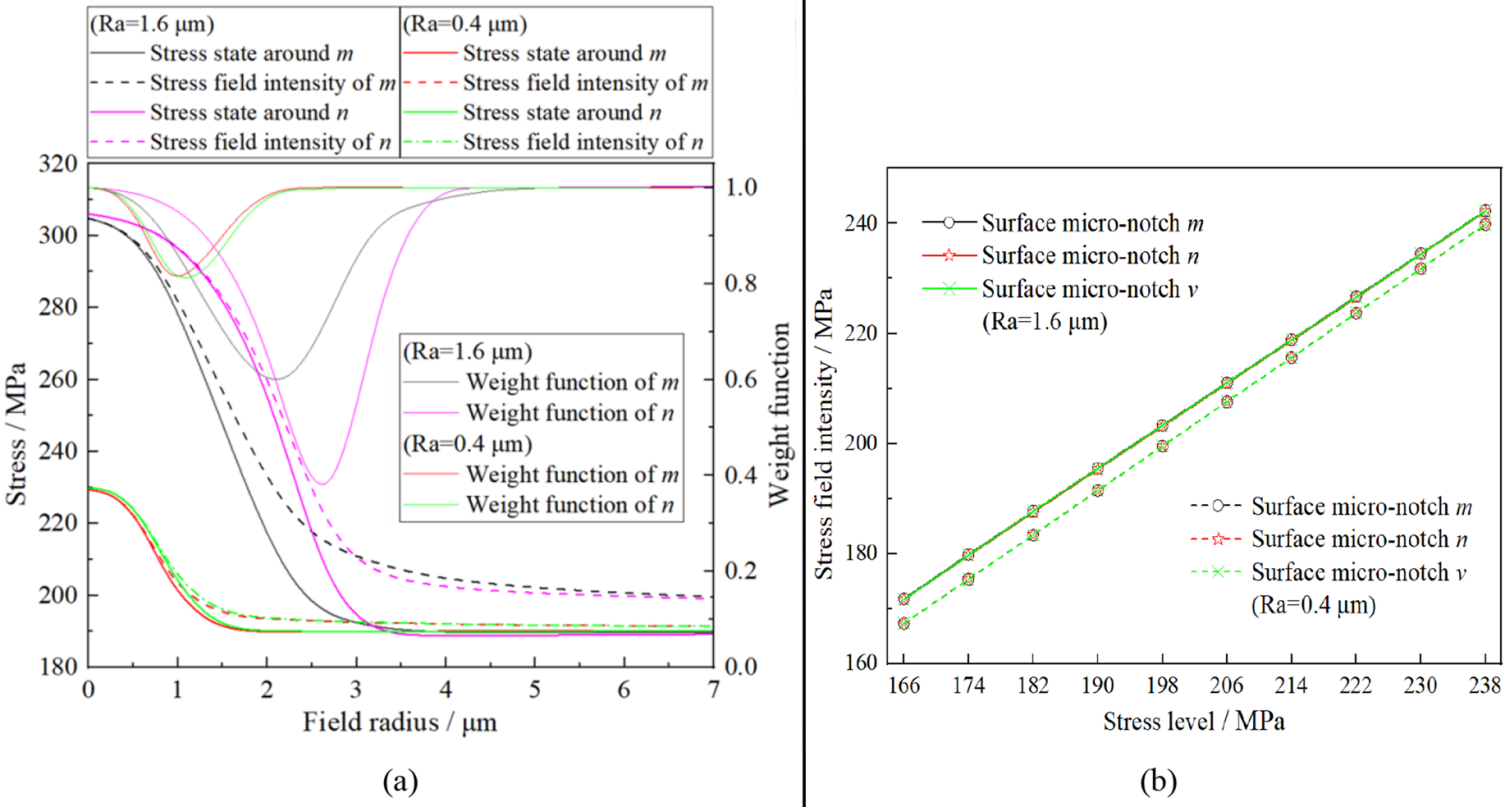
Calculation of fatigue damage parameter by the traditional stress field intensity approach with different surface roughness: (**a**) Stress field analyses around different micro-notches; (**b**) Stress field intensities of different micro-notches with different stress levels.

Similarly, taking planar plate specimen as an example, the stress field intensity of the surface micronotch can be calculated by Eqs. (3) and (4). In this case, the von Mises stress is taken as the equivalent stress. According to the FEM results shown in Fig. 8a, the stress field intensity of micronotches *m* and *n* with different surface roughnesses can be obtained by the traditional approach, when the field radius of Ω takes different values, as shown in Fig. 9a. To obtain the ultimate effective stress we need by the traditional method, the radius of Ω should be defined in advance. Based on the research result of Shang^45^, for the plane stress situation, the radius of Ω is calculated by Eq. (5) with the assumption that the shape of Ω can be treated as a circle.

5 $$r_{\max } = 0.2038 - 0.0242K_{{\text{t}}}$$

Although the surface micronotch is spherical, its stress state has central symmetry around the notch root, which is similar to that of the plane stress situation. For this reason, Eq. (5) can also be used to approximatively calculate the radius of Ω for a surface micronotch approximatively. According to the stress states around different surface micronotches, the stress concentration factors *K*_t_ of micronotches with surface roughnesses of 1.6 and 0.4 take values of 1.60 and 1.21, respectively. Calculated by Eq. (5), the radii of the fatigue damage failure regions for surface roughnesses of 1.6 μm and 0.4 μm can be obtained as 160 μm and 170 μm, respectively. In addition, based on the initial definition of the fatigue failure region, its radius was several grain sizes in extent. For micrograin size numbers 4–8 commonly used in engineering, the radius of Ω can take the value of 20–100 μm. However, our research (seen in Fig. 9a) has shown that the stress field intensity would be almost unchanged as the radius is large enough, *r*_max_ ≈ 4 μm for surface roughness of 1.6 μm and *r*_max_ ≈ 2 μm for surface roughness of 0.4 μm. Therefore, the values of stress field intensity are the same based on the two definition criteria of Ω.

Calculated by the traditional approach, the stress field intensities of different micronotches with different stress levels are illustrated in Fig. 9b. In Fig. 9b, the same as the maximum stress criterion, there is almost no difference among the stress field intensities of micronotches *m*, *n* and *v* under the same stress level, which can not explain the phenomenon in fatigue test, i.e., the three conclusions in Section “Experimental procedure and results”. In addition, the stress field intensity of the surface micronotch calculated by the traditional approach is almost equal to the corresponding nominal stress. Considering the existence of stress concentration at the surface micronotch, from a numerical point of view, the computational result of the traditional approach is obviously unreasonable. According to this inference, a smaller radius of Ω should be considered during the calculation of the stress field intensity. As shown in Fig. 9a, the value of the stress field intensity with a smaller radius fluctuates, and the computational result has a strong relationship with the size of Ω, which is unreasonable for the fuzzy definition of Ω. In other words, the stress field intensity calculated by the traditional approach has a strong fluctuation as the radius of Ω can not be clearly defined. Moreover, there is no accessible sign of the field intensity curve at the boundary of Ω in this case. This means that the boundary line of the field intensity curve is artificially defined in this case, which is unreasonable considering the continuity of fatigue damage in the material. In conclusion, the traditional approach is also not applicable to the calculation of fatigue damage parameters for the surface micronotch on the order of magnitude of roughness size.

### Revised stress field intensity approach

Furthermore, the main reason for the defect of the traditional approach can be interpreted as the peak stress of the surface micronotch was dealt with the other stress around the micro-notch equivalently in the calculation process, although the weight function was employed to adjust the primary and secondary sequences among them. From the initial definition of the weight function in the traditional approach, it is generally acknowledged that the value of weight function is mainly determined by two important parameters of a reference point, i.e., stress gradient and distance from the micronotch root, under the certain loading conditions. For a notch of conventional size, there is no dissidence to the value of this parameter (*φ*(*r* *>* 0) < 1) and the weight function is a generalized monotonically decreasing function about *r* in the fatigue damage failure region. However, for the surface micronotch in the order of the magnitude of roughness size, the trend of *r* has a little impact on the value of the weight function, because of the very small stress concentration region. In this case, the value of weight function is influenced only by the stress gradient. Therefore, apart from a very small region with a considerable stress gradient around the micronotch root, the value of weight function in great portion of the fatigue damage failure region tends to 1 (shown in Fig. 9a), which means the weight function can no longer adjust the primary and secondary sequences among the stresses at different points in this condition. To illustrate this problem, by rewriting Eqs. (3) and (4), the traditional approach can be simplified to the stress average criterion when *φ*(*r*-*θ*) = 1, as shown in Eq. (6)^48^.

6 $$\begin{aligned} \sigma_{FI} & = \frac{1}{V}\int\limits_{\Omega } {\sigma_{r - \theta } } \times \left[ {1 - \left| {\frac{1}{{\sigma_{\max } }} \times \frac{{{\text{d}}\sigma_{r - \theta } }}{{{\text{d}}r}}} \right|r(1 + \sin \theta )} \right]{\text{d}}v \\ & = \frac{1}{V}\int\limits_{\Omega } {\sigma_{r - \theta } } {\text{d}}v - \frac{1}{V}\int\limits_{\Omega } {\left| {\frac{{\sigma_{r - \theta } }}{{\sigma_{\max } }} \times \frac{{{\text{d}}\sigma_{r - \theta } }}{{{\text{d}}r}}} \right|r(1 + \sin \theta ){\text{d}}v} \\ \end{aligned}$$

However, based on the fatigue failure mechanisms of the notched components, there are obvious differences in the damage processes of different points as an effect of the stress gradient. However, it is difficult to directly measure or accurately identify the damage processes by the existing technology. To solve this problem, a revised stress field intensity approach accounting for the fundamental principles of the traditional approach was introduced in this part, in which the continuous damage process around the surface micronotch of the specimen was analyzed. Different from the traditional approach, it is considered in the revised approach that the damage amount of the stress peak point plays a leading role in fatigue damage of the surface micronotch. Under this premise, the stresses of the other points around the surface micronotch only play a corrective role in the fatigue damage of the peak point.

#### Definition of correction parameter for revised approach

The revised approach notes that fatigue failure of the surface micronotch is simultaneously affected by the peak stress and stress distribution around the micronotch root. Based on the original assumption of the traditional approach, the function form of *φ*(*r*-*θ*) and parameters contained in this revised approach were improved. Based on the inference above, two improvements for the traditional approach were expressed as follows:

(1) Correction for fatigue damage failure region

Considering the peak stress as the principal fatigue damage parameter, the other stresses of the fatigue damage failure region will act as a correction function. The revised fatigue damage parameter can be expressed as

7 $$\sigma_{FI} = \sigma_{\max } + \varsigma (\Omega )$$

where $\varsigma (\Omega )$ is a new correction parameter caused by the effect of all stresses in Ω. New research shows that the value of the correction parameter in one point is associated with its stress difference with the peak stress. Using statistical average, the correction parameter of all points in the fatigue failure region can be expressed as

8 $$\varsigma (\Omega ) = \frac{{\int\limits_{\Omega } {\left( {\sigma_{r - \theta } - \sigma_{\max } } \right) \times \varphi (r - \theta ){\text{d}}v} }}{{\int\limits_{\Omega } {\varphi (r - \theta ){\text{d}}v} }}$$

In Eq. (8), the value ranges of the two parameters can be obtained based on the FEM, 0 ≤ *φ*(*r* − *θ*) ≤ 1 and (*σ*_*r* − *θ*_ − *σ*_max_) ≤ 0. Accordingly, it is obvious that the correction parameter $\varsigma (\Omega )$ is negative, which means that the failure effect of the peak stress at the micronotch will be weakened with the consideration of other stresses in Ω. In other words, the other point in the fatigue failure region has an adverse effect on the fatigue failure of the peak stress point. In addition, for ideal smooth plain specimens, the stresses in Ω are the same and the parameter (*σ*_*r*-*θ*_ − *σ*_max_) will take the value of 0, i.e., $\varsigma (\Omega ) = 0$ calculated by Eq. (8). Therefore, the specimen damage process can be directly represented by any stress at the gauge section of the smooth plain specimen. In other words, any stress of the smooth plain specimen can be assessed separately without interference with each other, which is widely recognized.

(2) Revision of the weight function

To overcome the defect of the traditional weight function, a new form of weight function is proposed in this section. First, different from the linear relationship adopted in the traditional approach, the form of a power function or exponential function is considered for parameter *r* to address the surface micronotch on the order of magnitude of roughness size. When the power function is employed, the original assumption of weight function *φ*(0) = 1 will not be supported. For this reason, the exponential function is used in the new weight function, before which nondimensional of treatment for the parameter *r* should be done. Considering that the radius of the fatigue damage failure region is associated with the size of several gains, the average diameter (*R*) of the material grain is used and Eq. (4) can be rewritten as

9 $$\varphi (r - \theta ) = \left| {\frac{{{\text{d}}\sigma_{r - \theta } }}{{{\text{d}}r}} \times \frac{x}{{\sigma_{\max } }}} \right|^{{\frac{r\cos \theta }{R}}}$$

where *x* is an undetermined coefficient to unify the dimension of the weight function. In the present study, experimental verification shows that the coefficient *x*/*σ*_max_ can be defined as the reciprocal of the maximum stress gradient *max*(d*σ*_*r*-*θ*_/d*r*) around the surface micronotch and the new form of weight function can be expressed by

10 $$\varphi (r - \theta ) = \left| {\frac{{{\text{d}}\sigma_{r - \theta } }}{{{\text{d}}r}} \times \frac{1}{{\max (d\sigma_{r - \theta } /dr)}}} \right|^{{\frac{r\cos \theta }{R}}}$$

#### Simplification of the revised stress field intensity approach

Similar to traditional approaches, the revised approach needs the analysis of the stress state near the surface micronotch at the volume or area level with a lot of calculation. To solve the problem, a simplified method is required to transform the integral form on a block or surface element into a multiple integral form on a special line.

**Figure 10:**
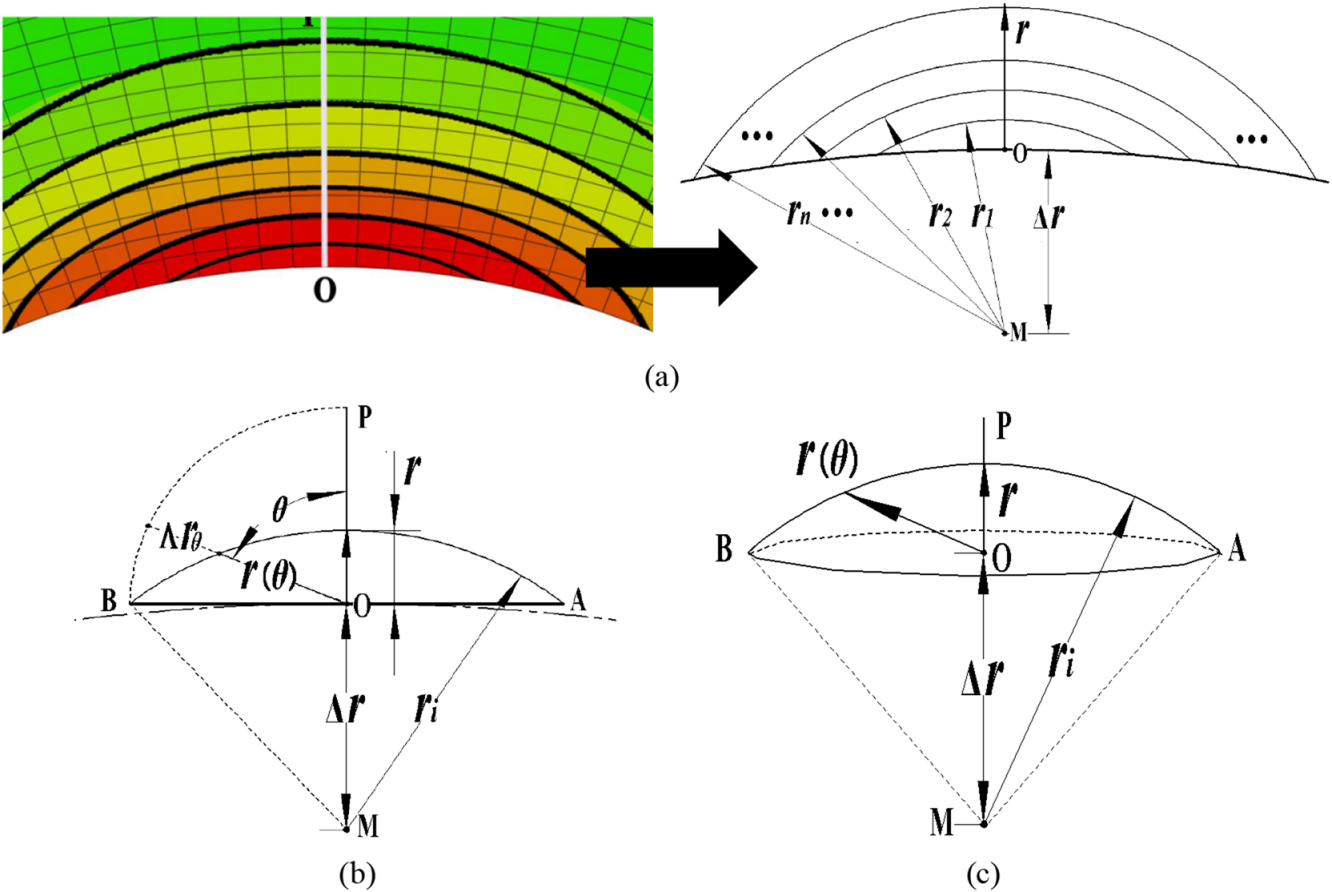
Simplifications of stress contour line near notch root: (**a**, **b**) Regular circular notch in plane stress; (**c**) Spherical notch.

Taking the simple plane stress problem as an example, the stress contour line aperture of the nephogram around the regular circular notch is shown in Fig. 10a, where “O” is the notch root. As shown in Fig. 10a, the stress distributions around the notch root have a strong regularity, i.e., a regular circular distribution. Based on this characteristic, it can be assumed that the stress contour lines near the notch root are concentric circles with a center M, just as shown in Fig. 10a, where *r*_*i*_ is the radius of the stress contour line, and △*r* is the distance between points “M” and “O”.

As shown in Fig. 10b, the simplifications of the stress contour line near the notch root show that the stress field states on the same contour line are equivalent, except for the deviation angles *θ* and distances *r* of different reference points. Therefore, it is reasonably practicable to choose the stress state of the point on the symmetrical line for the correction parameter calculation of the corresponding contour line. To make this come true, the coupling relation of *θ* and *r* should be analyzed first. On the basis of the geometrical analysis in Fig. 10b, the boundary conditions of function *r*(*θ*), in which significant trigonometric relationship exists, can be obtained

11 $$\left\{ {\begin{array}{*{20}l} {1)} \hfill & {r(0) = r} \hfill \\ {2)} \hfill & {r(\frac{\pi }{2}) = \sqrt {r_{i}^{2} - \Delta r_{{}}^{2} } } \hfill \\ {3)} \hfill & {r^{{\prime }} (0) = 0} \hfill \\ {4)} \hfill & {r_{i} = r + \Delta r} \hfill \\ \end{array} } \right.$$

Based on the inference above, the simplest form among infinite functions derived by the boundary conditions mentioned above can be obtained

12 $$r(\theta ) = \left( {\sqrt {r_{{}}^{2} + 2 \times r \times \Delta r} - r} \right) \times \left( {1 - \cos \theta } \right) + r$$

Moreover, as the outline radian of the notch is far greater than that of the stress contour line, the outline of the notch is simplified to a straight line, by which the correction parameter of the stress contour line with distance *r* from the peak stress point is defined

13 $$\varsigma (r) = \frac{{\int_{0}^{{\frac{\pi }{2}}} {\left( {\sigma_{r} - \sigma_{\max } } \right) \times \left| {\frac{{{\text{d}}\sigma_{r} }}{{{\text{d}}r}} \times \frac{1}{{\max (d\sigma_{r} /dr)}}} \right|^{{\frac{r\left( \theta \right)\cos \theta }{R}}} {\text{d}}\theta } }}{{\int_{0}^{{\frac{\pi }{2}}} {\left| {\frac{{{\text{d}}\sigma_{r} }}{{{\text{d}}r}} \times \frac{1}{{\max (d\sigma_{r} /dr)}}} \right|^{{\frac{r\left( \theta \right)\cos \theta }{R}}} {\text{d}}\theta } }}$$

where *σ*_*r*_ is stress of the stress contour line with the distance *r* from the peak stress point.

In summary, with integral calculation for stress contour lines near the notch root, core formulae of the proposed method l are obtained

14 $$\left\{ {\begin{array}{*{20}l} {\varsigma (\Omega ) = \frac{{\int_{0}^{{r_{\max } }} {\int_{0}^{{\frac{\pi }{2}}} {\left( {\sigma_{r} - \sigma_{\max } } \right) \times \left| {\frac{{{\text{d}}\sigma_{r} }}{{{\text{d}}r}} \times \frac{1}{{\max (d\sigma_{r} /dr)}}} \right|^{{\frac{r\left( \theta \right)\cos \theta }{R}}} {\text{d}}\theta } {\text{d}}r} }}{{\int_{0}^{R} {\int_{0}^{{\frac{\pi }{2}}} {\left| {\frac{{{\text{d}}\sigma_{r} }}{{{\text{d}}r}} \times \frac{1}{{\max (d\sigma_{r} /dr)}}} \right|^{{\frac{r\left( \theta \right)\cos \theta }{R}}} {\text{d}}\theta } {\text{d}}r} }}} \hfill \\ {\sigma_{FI} = \sigma_{\max } + \varsigma (\Omega )} \hfill \\ \end{array} } \right.$$

For the spherical notch used in this paper, the stress contour line in plane stress will be replaced with a stress contour plane. Similarly, core formulae of the correction parameter can be obtained for the spherical notch by geometric morphology analysis (Fig. 10c).

15 $$\varsigma (r) = \frac{{\int_{0}^{{\frac{\pi }{2}}} {\left( {2\pi \times r\left( \theta \right)\sin \theta } \right) \times \left( {\sigma_{r} - \sigma_{\max } } \right) \times \left| {\frac{{{\text{d}}\sigma_{r} }}{{{\text{d}}r}} \times \frac{1}{{\max (d\sigma_{r} /dr)}}} \right|^{{\frac{r\left( \theta \right)\cos \theta }{R}}} {\text{d}}\theta } }}{{\int_{0}^{{\frac{\pi }{2}}} {\left( {2\pi \times r\left( \theta \right)\sin \theta } \right) \times \left| {\frac{{{\text{d}}\sigma_{r} }}{{{\text{d}}r}} \times \frac{1}{{\max (d\sigma_{r} /dr)}}} \right|^{{\frac{r\left( \theta \right)\cos \theta }{R}}} {\text{d}}\theta } }}$$

16 $$\varsigma (\Omega ) = \frac{{\int_{0}^{{r_{\max } }} {\int_{0}^{{\frac{\pi }{2}}} {\left( {2\pi \times r\left( \theta \right)\sin \theta } \right) \times \left( {\sigma_{r} - \sigma_{\max } } \right) \times \left| {\frac{{{\text{d}}\sigma_{r} }}{{{\text{d}}r}} \times \frac{1}{{\max (d\sigma_{r} /dr)}}} \right|^{{\frac{r\left( \theta \right)\cos \theta }{R}}} {\text{d}}\theta } {\text{d}}r} }}{{\int_{0}^{R} {\int_{0}^{{\frac{\pi }{2}}} {\left( {2\pi \times r\left( \theta \right)\sin \theta } \right) \times \left| {\frac{{{\text{d}}\sigma_{r} }}{{{\text{d}}r}} \times \frac{1}{{\max (d\sigma_{r} /dr)}}} \right|^{{\frac{r\left( \theta \right)\cos \theta }{R}}} {\text{d}}\theta } {\text{d}}r} }}$$

According to the simplification of stress contour lines around the surface micronotch, the integral form on the fatigue failure region for the correction parameter is replaced by a multiple integral form on the outline symmetrical line of the notch. Different from the traditional form, only the stresses on the symmetrical plane of micronotch are needed in the computational process of the simplified approach, which can reduce the quality requirement of the FEM mesh and simplify the later integral computational process.

**Figure 11:**
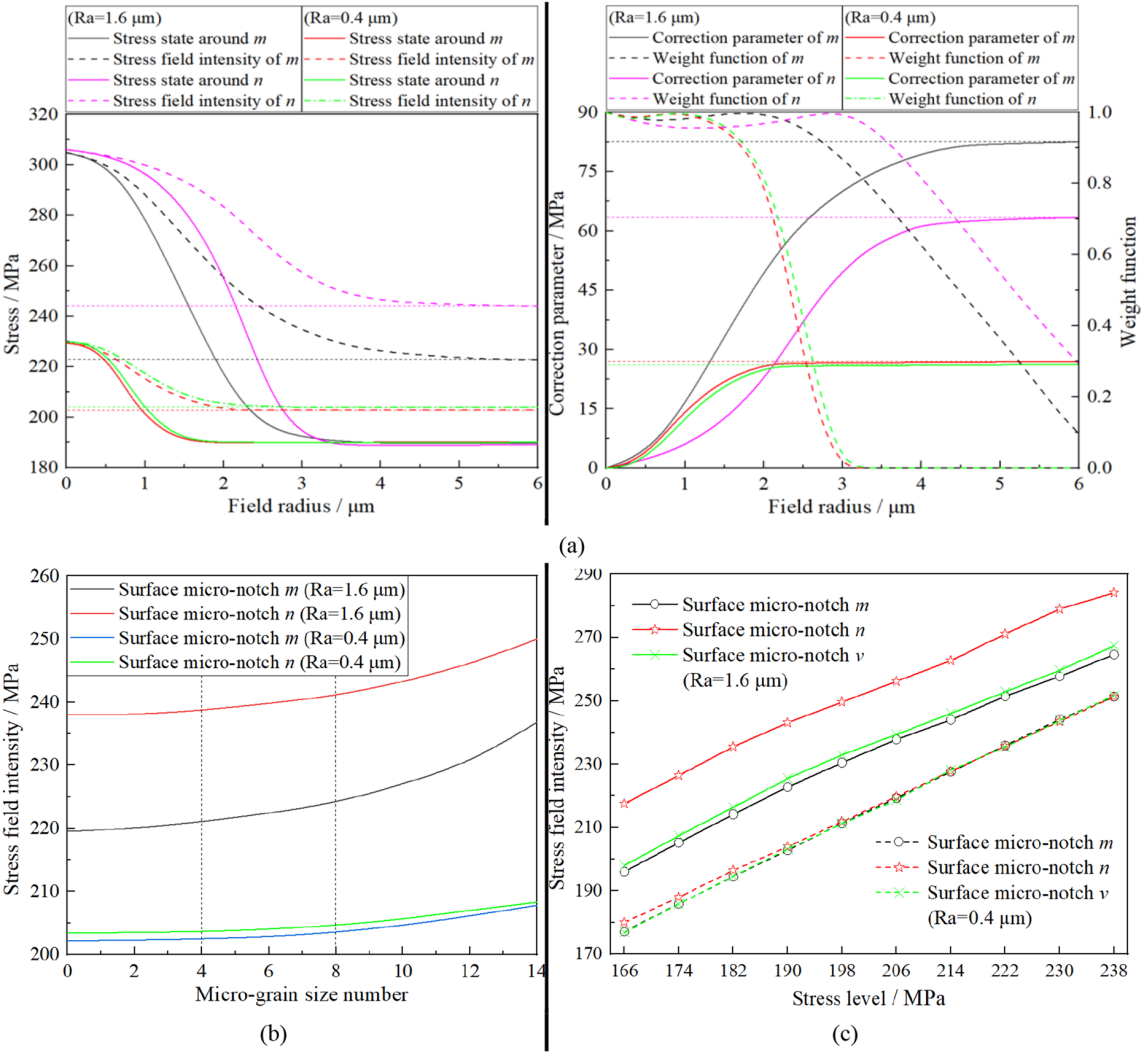
Calculation of fatigue damage parameter by the revised approach with different surface roughness: (**a**) Stress field analyses around different micro-notches; (**b**) Grain size effect on computational result; (**c**) Stress field intensities of different micro-notches under the different stress levels.

According to the FEM results shown in Fig. 8a, the stress field intensity of micronotches *m* and *n* with different surface roughnesses can be calculated by the new proposed model for different values of the field radius of the fatigue failure region, just as plotted in Fig. 11a. As shown in Fig. 11a, with increasing field radius, the correction effects of points in Ω decrease significantly. In other words, the correction parameter near the surface micronotch will tend to be immutable when *r* is large enough, which means that the calculation result of the stress field intensity will reach its accurate and objective result correspondingly. Thus the revised stress field intensity approach weakens the effect of *r* and the definition difficulty of Ω is fundamentally overcome. The computational result was obtained based on the fact that the micrograin size number was 6. To analyze the effects of micrograin size number, the stress field intensities of micronotches *m* and *n* with different surface roughnesses were calculated using the new revised approach with different grain sizes, micrograin size numbers selected from 0 to 14, as shown in Fig. 11b. It is obvious that the stress field intensity obtained by the revised approach has a low sensitivity to the micrograin size number. Especially for the common micrograin size numbers 4–8, the fluctuation range of the computational results for different conditions is within the range of 0.5–1.5%.

Calculated by the revised approach, the stress field intensities for different micronotches are illustrated in Fig. 11c. There is an obvious difference among the stress field intensities of micronotches *m*, *n* and *v* with the higher surface roughness (*Ra* = 1.6 μm) , as shown in Fig. 11c, while there is almost no difference with the lower surface roughness (*Ra* = 0.4 μm). From the visual viewpoint, the computational result of the revised approach conforms to the phenomen in the fatigue test, i.e., the three conclusions in Section “Experimental procedure and results”.

## Discussion on the usability of the improved approach

### Explanation for the experimental results (qualitative analysis of the improved approach)

According to the stress field intensities of different micronotches under different stress levels (calculated by the revised approach proposed in Section “Simplification of the revised stress field intensity approach”), the following conclusions can be drawn:

1. By comparing the stress field intensities on various criteria in Fig. 11c, it is obvious that, for the same nominal stress, the stress field intensity of surface micronotch decreases when the surface roughness decreases from 1.6 μm to 0.4 μm. This means the fatigue damage parameter of the specimen with surface roughness (*Ra* = 1.6 μm) is higher than that of the specimen with the surface roughness (*Ra* = 0.4 μm) under the same stress level. Therefore, the rough specimen is more likely to experience fatigue failure than the polished specimen in the same loading environment, i.e., the lower fatigue strength for the rough specimen.
2. Based on the polished region, the stress field intensities of the surface micronotch *m*, *v* and *n* increase in turn with the same nominal stress, as shown in Fig. 11c. In addition, differences among the stress field intensities of different surface micronotches will be obscure or unclear with decreasing surface roughness. It can be inferred from the conclusion above that the percentage of fatigue damage parameter decreased by polishing the planar surface, curved surface and corner region increases in turn with the same nominal stress. Therefore, for planar plate specimens, it is more useful to reduce the fatigue damage parameter (i.e., promoting the fatigue strength of the specimen) by polishing the corner region of the specimen than the planar surface under the same stress level.

**Figure 12:**
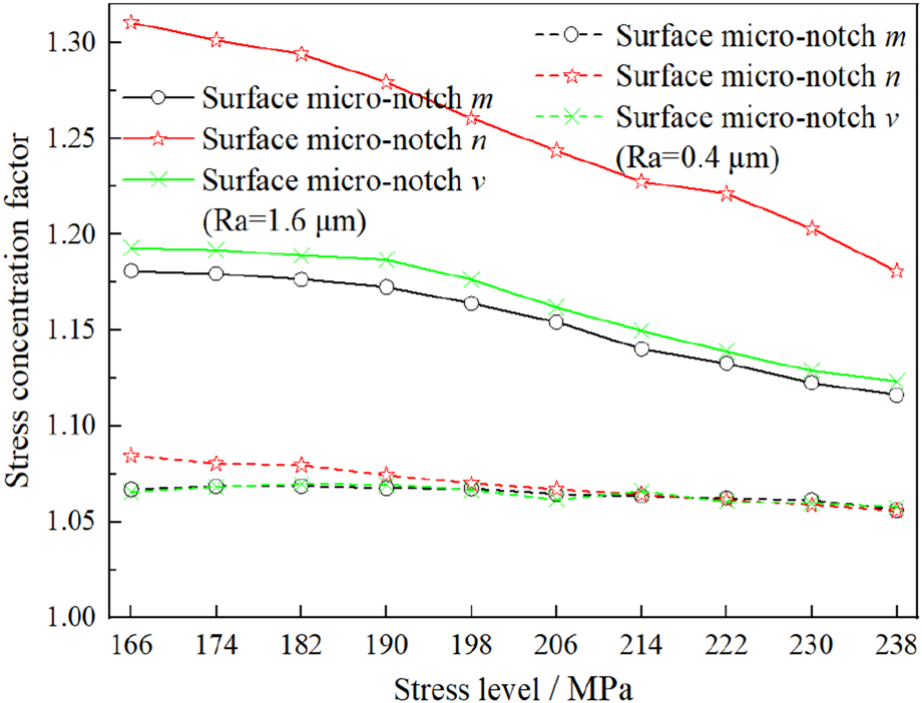
Stress concentration factors of different micro-notches under the different stress levels. (Calculated by revised approach).

The two conclusions above obtained by the revised approach conform to he three experimental phenomena in Section “Experimental procedure and results”. Moreover, Fig. 4b shows another implicit phenomenon in which the influence of the surface roughness on the fatigue limit of the specimen declines with increasing stress level. To explain the experimental phenomenon by the revised approach, the stress concentration factors of different micronotches under the different stress levels can be calculated by the revised approach, shown in Fig. 12. It is obvious that the stress concentration factor of surface micronotch decreases with increasing stress level applied to the specimen, which can explain the experimental phenomenon precisely.

### Quantitative analysis of the improved approach

**Figure 13:**
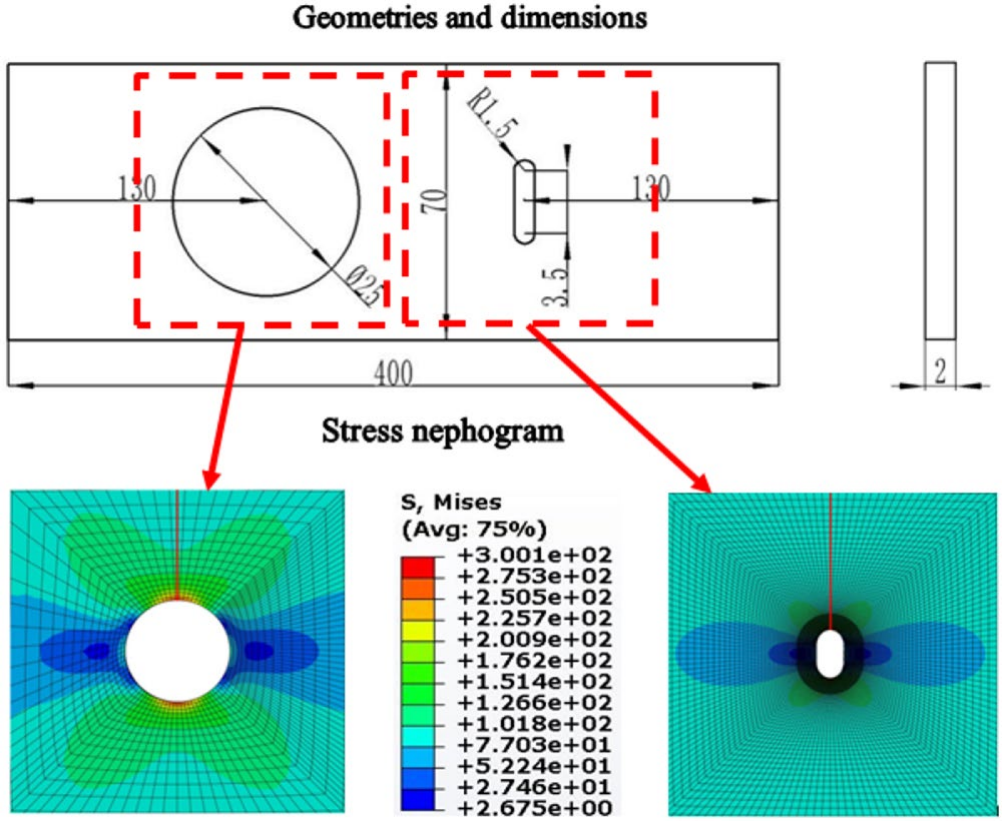
Geometries and dimensions of DH-A specimen and stress nephogram of the two notch roots under stress level 80 MP. (Unit: mm).

**Figure 14:**
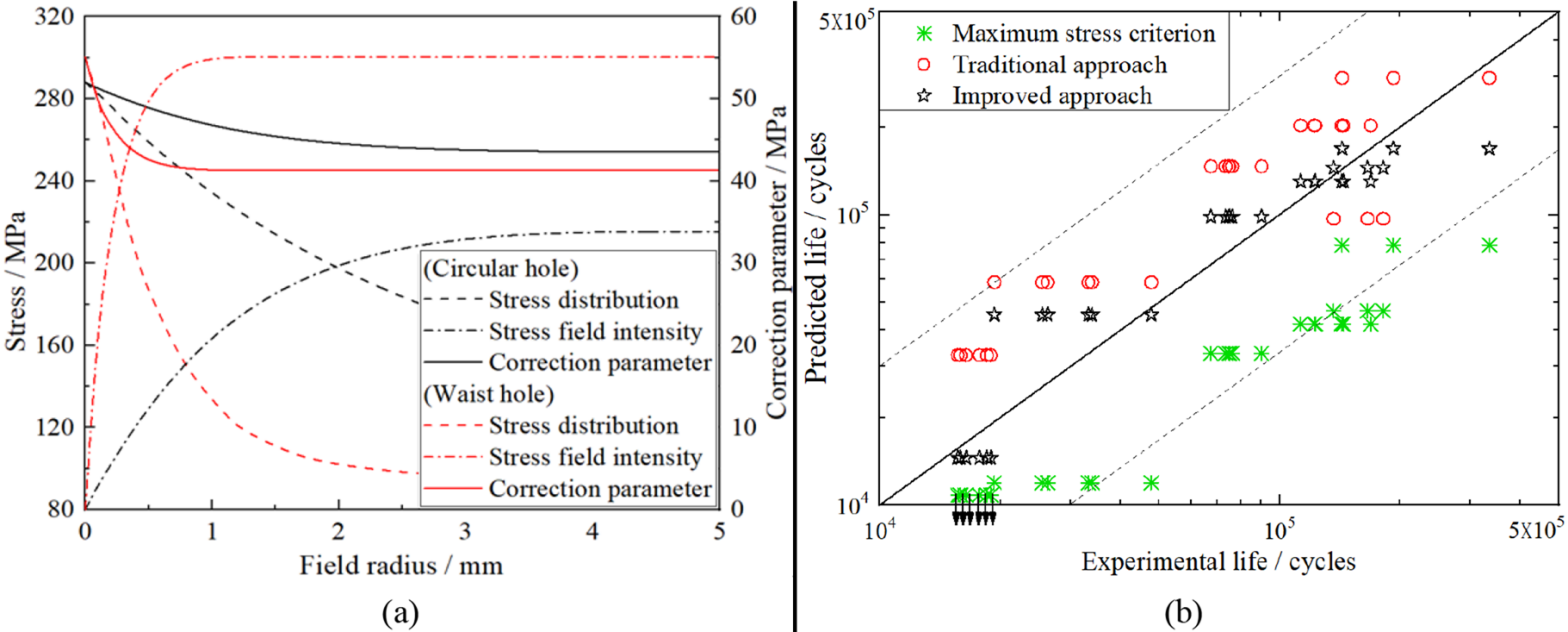
Analysis of fatigue problems for the notch in conventional size: (**a**) Stress field analysis for different holes by the revised approach; (**b**) Accuracy comparison of the three approaches for different notched specimens.

To demonstrate the accuracy of the revised approach quantitatively, the DH-A specimen (made from 2A12 aluminum alloy, AlCu4Mg1 in ISO) in Reference^49^ has been subjected to stress field intensity calculation. The geometries and dimensions of DH-A are shown in Fig. 13a. In Fig. 13a, it is obvious that there are two dangerous sites on DH-A, named the waist hole and circular hole. In our study, ABAQUS was chosen to calculate the stress distribution around the two holes. Parameter information of 2A12 aluminum alloy needed in FEM can be obtained from the Reference^49^. When the stress level applied on the specimen is 80 MPa, the stress states around the two holes are illustrated in Fig. 13b, based on which the stress field parameters with different field radii for the two holes are shown in Fig. 14a. As shown in Fig. 14a, it is obvious that, similar to the surface micronotch in the order of magnitude of roughness size (Fig. 11a), the stress field parameters calculated by the revised approach for the notch of conventional size tend to be immutable when *r* is large enough, which means that the calculation result of stress field intensity will reach its accurate and objective result correspondingly.

Similarly, the stress field parameters of the DH-B, DN-A and DN-B specimens can be obtained by the proposed revised approach. On the basis of the S–N curve of the 2A12 aluminum alloy^49^, fatigue lives of different specimens can be obtained. In addition, to contrast the accuracy of different approaches for fatigue strength assessment, the traditional approach and maximum stress criterion were also employed to analyze the experimental data. The comparison result of the three approaches is illustrated in Fig. 14b. Figure 14b shows that, for the four kinds of notches of conventional size, the revised approach has a narrower error band than the other two approaches, especially for the comparison result with the maximum stress criterion, which means the revised approach has a satisfactory accuracy from the perspective of quantitative analysis.

## Conclusions

In this paper, different underlying physical mechanisms of the roughness effect at different regions of specimens were studied and a revised stress field intensity approach for a fatigue strength assessment of microsized notches was proposed as theoretical support. The following conclusions can be drawn:

1. Analyzing the effect of surface roughness on material fatigue performance also explores the fatigue characteristics of notch structures. Fatigue failure for notch structures with stress concentrations will not occur at a single peak point of stress but in a certain high stress region due to the effects of the stress/strain gradient. From this point of view, the new method represented by the stress field strength method, with the effective damage parameter set to be the stress field intensity for the high stress region, is more effective in dealing with the fatigue of the notch structure (i.e., the effect of surface roughness) compared with traditional methods such as the nominal stress method and local stress–strain method. In addition, to adapt to the surface micronotch on the order of magnitude of roughness size, a revised stress field intensity approach was built in this paper, the accuracy and stability of which were verified by a variety of experimental data.
2. Surface roughness is a significant influencing factor on the fatigue strength of specimens. On the one hand, the fatigue limit of the specimen increases with the improvement of specimen surface quality. This can be interpreted as follows: in the same load environment, the stress level in the high stress region around the surface micronotch decreases with the improvement of specimen surface quality, i.e., with a lower effective damage parameter (stress field intensity). On the other hand, the fatigue limit of the specimen can be affected by its polished regions, and the possibilities of corner fracture, curved surface fracture and planar surface fracture decrease in turn in the same load environment. For specimens with uniform surface roughness, the peak stresses of the surface micronotches at the corner region, curved surface region and planar region are approximate in the same load environment, which cannot explain the effect of the polished region. When the revised stress field intensity approach is employed, it can be seen that there are significant differences in the effects of the stress gradient in the high stress region around different region surface micronotches. Due to the different effects of the stress gradient at different polished regions, the effective damage parameters (stress field intensities) of the surface micronotch at the corner region, curved surface region and planar region significantly decrease in turn in the same load environment, consistent with the variation law of fatigue limits.

## Supplementary Information

Supplementary Information.
