# Supplementary material for: Surface roughness effect on fatigue strength of aluminum alloy using revised stress field intensity approach
Source: Sci Rep. 2021 Sep 29;11:19279. doi: 10.1038/s41598-021-98858-0 (PMC8481500; doi:10.1038/s41598-021-98858-0)
Supplement: Supplementary file 1 — Supplementary Information. [file 41598_2021_98858_MOESM1_ESM.docx]

**Appendix 1：Morphology of raw material**


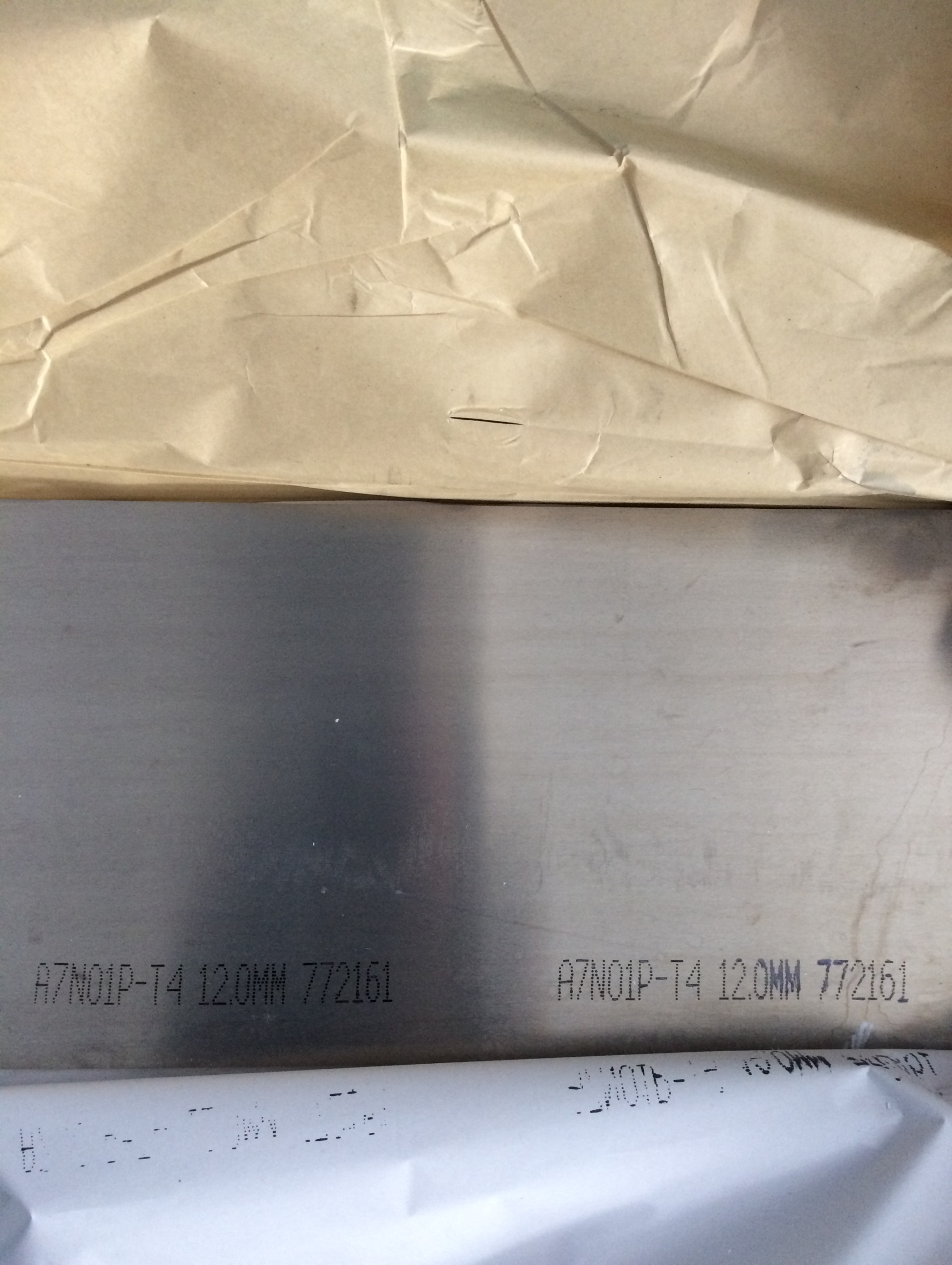


**Appendix 2：Calibration certificate of testing machine**


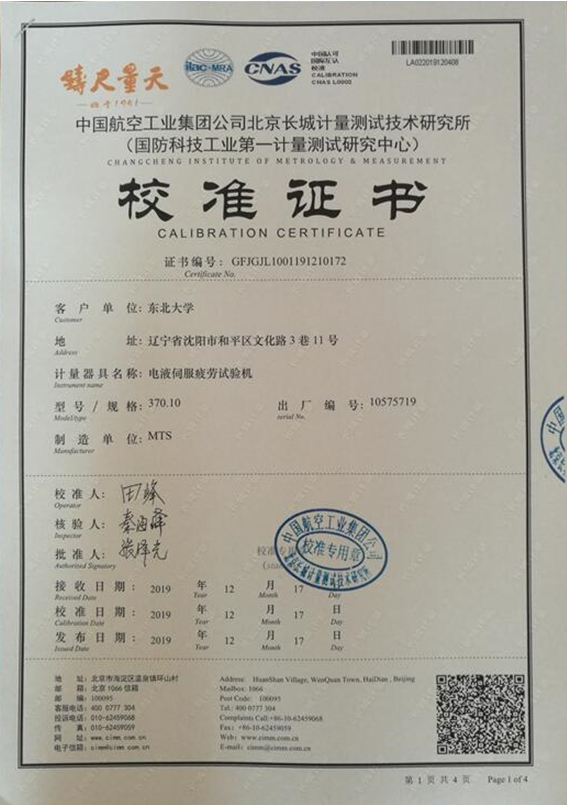


MTS hydraulic servo fatigue testing machine


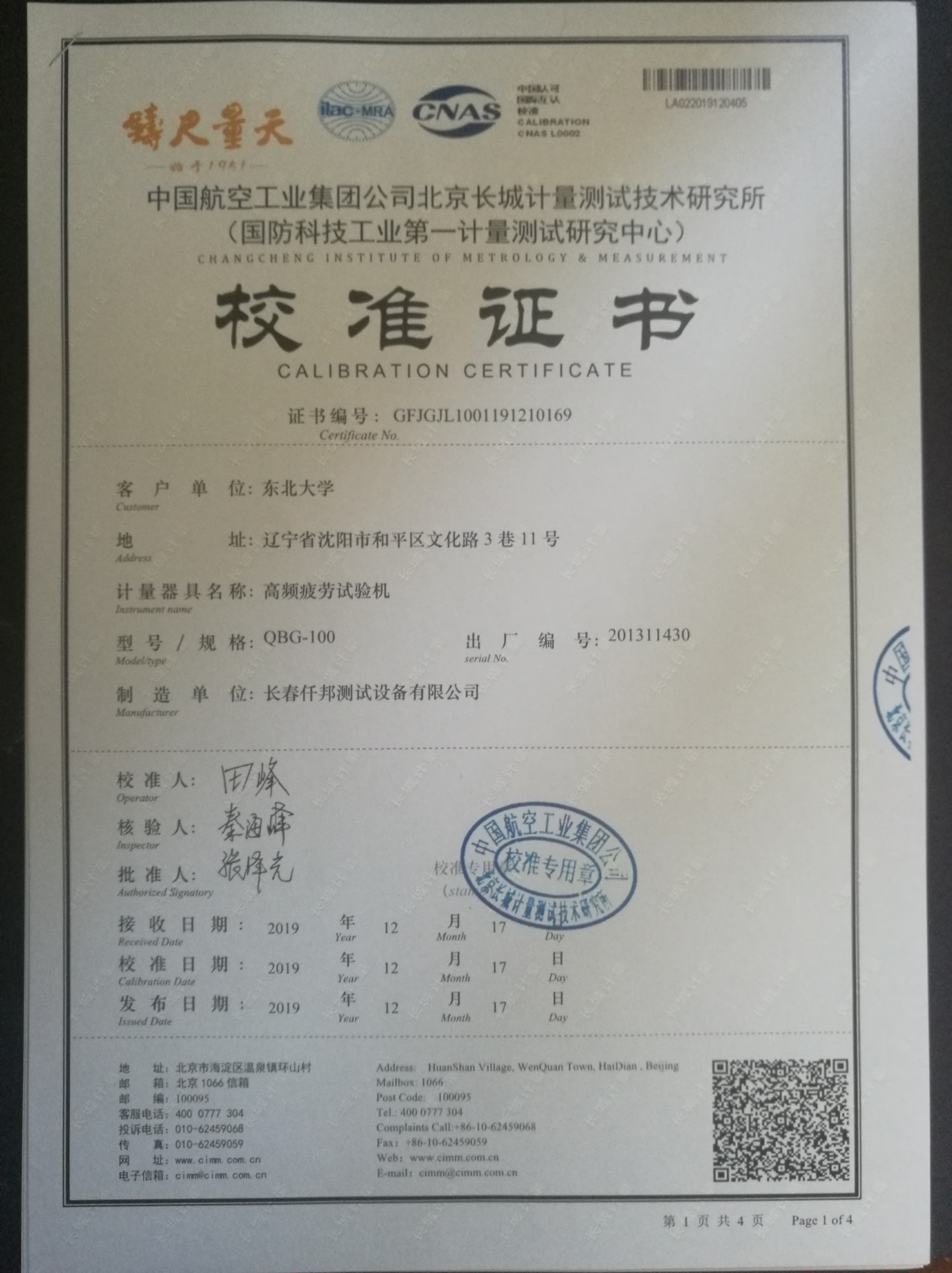


QBG electromagnetic resonance high frequency fatigue testing machine
